# A Novel Surface Modification Strategy via Photopolymerized Poly-Sulfobetaine Methacrylate Coating to Prevent Bacterial Adhesion on Titanium Surfaces

**DOI:** 10.3390/ma14123303

**Published:** 2021-06-15

**Authors:** Aydin Gülses, Antonia Lopar, Martha Es-Souni, Marie Emmert, Mona Es-Souni, Eleonore Behrens, Hendrik Naujokat, Kim Rouven Liedtke, Yahya Acil, Jörg Wiltfang, Christian Flörke

**Affiliations:** 1 Department of Oral and Maxillofacial Surgery, Faculty of Medicine, Christian-Albrecht University, UKSH, 24105 Kiel, Germany; antonia_lopar@yahoo.de (A.L.); emmert.marie@googlemail.de (M.E.); behrens@mkg.uni-kiel.de (E.B.); yahya.acil@uksh.de (Y.A.); joerg.wiltfang@uksh.de (J.W.); 2 Department of Orthodontics, Faculty of Dentistry, Christian-Albrecht University, UKSH, 24105 Kiel, Germany; es-souni@kfo-zmk.uni-kiel.de (M.E.-S.); monaessouni@gmail.com (M.E.-S.); 3 Department of Orthopedics, Faculty of Medicine, Christian-Albrecht University, UKSH, 24105 Kiel, Germany; kimrouven.liedtke@uksh.de

**Keywords:** titanium, antibacterial, polysulfobetaine methacrylate

## Abstract

Recent investigations on the anti-adhesive properties of polysulfobetaine methacrylate (pSBMA) coatings had shown promising potential as antifouling surfaces and have given the impetus for the present paper, where a pSBMA coating is applied via photopolymerization on a macro-roughened, sandblasted, and acid-etched titanium implant surface in order to assess its antifouling properties. Current emphasis is placed on how the coating is efficient against the adhesion of Enterococcus faecalis by quantitative assessment of colony forming units and qualitative investigation of fluorescence imaging and scanning electron microscopy. pSBMA coatings via photopolymerization of titanium surfaces seems to be a promising antiadhesion strategy, which should bring substantial benefits once certain aspects such as biodegradation and osseointegration were addressed. Additionally, commercial SAL-titanium substrates may be coated with the super-hydrophilic coating, appearing resistant to physiological salt concentrations and most importantly lowering E. faecalis colonization significantly, compared to titanium substrates in the as-received state. It is very likely that pSBMA coatings may also prevent the adhesion of other germs.

## 1. Introduction

Besides cell factors release at the implant recipient site, the non-specific protein adsorption or the so called “biofouling” is one of the first primary steps of foreign body reaction during placement of an implant. Since the 1950’s, titanium has been widely used in many fields of medicine due to its superior bio-compatibility, corrosion resistance, strength, and low modulus. However, infections secondary to medical implants remain challenging and are also not uncommon in patients with titanium medical implants [1]. Therefore, there is a growing interest on developing anti-biofouling surface treatment strategies for the prevention of bacterial adhesion and colonization, which represent important processes towards progression of infection.

In the literature, several antifouling strategies for titanium surfaces have been described, such as titanium nanotube coatings [2], modifications with nanoarrays [3], plasma immersion ion implantation onto the implant surface [4], antibiotic loaded coatings/and/or antimicrobial peptide deposition [5,6], as well as the use of bactericidal metal ions such as Ag, Cu, and Zn [7]. In addition to the above-mentioned strategies, applying a layer of physiologically inert polymer on the titanium surface has become one of the commonly used antifouling strategies in recent years [8]. In this respect, the inherent antiadhesive nature of the polymer suppresses bacterial interaction with the implant surface. In the literature, coatings with poly(ethyleneglycol) (PEG) [9,10] and PEG derivates (polyethylene oxide and pluronic F-127), nafion, [10,11,12], poly (oligo ethylene glycol) (POEG) [13], polycarbonate, zwitterionic polymers [14], and dextran have been described as effective anti-adhesion strategies for titanium surfaces [15,16].

A recent work performed coating of polysulfobetaine methacrylate (pSBMA) brushes directly onto the pore walls of NiTi nanotubes and yielded an anti-adhesive surface, which might be used for titanium implants [17]. Additionally, pSBMA has been suggested to be successfully grafted onto the pore walls of TiO_2_ films and presents a a versatile and environmentally friendly photo-grafting technique [17]. Moreover, pSBMA grafted films were proclaimed to improve the wetting angles of the pore walls, which leads to a three-dimensional nanocomposite, which is mechanically stable and particularly protein and thereby bacteria-repellent [17]. Therefore, it might be possible to selectively modify an implant surface with areas of high cell adhesion, where cell adhesion is required and has areas with diminished protein adhesion, where bacterial adhesion should be avoided. Preliminary investigations of the anti-adhesive properties of these coatings had shown their promising potential as antifouling surfaces and gave the impetus for the present paper, where a pSBMA coating is applied via photopolymerization on a macro-roughened, sandblasted, and acid-etched titanium implant surface in order to assess its antifouling properties. Current emphasis is placed on how the coating is efficient against the adhesion of Enterococcus faecalis.

## 2. Materials and Methods

### 2.1. Chemical Substances, Culture Vessels, and Bacteria

The chemical substances used were: 3-(Trimethoxysilyl)propyl methacrylate (TMSPMA, Sigma Aldrich, St. Louis, MO, USA); [2-(Methacryloyloxy)ethyl]dimethyl-(3-sulfopropyl)ammonium hydroxide 95% (SBMA, Sigma Aldrich); 1-phenyl-1,2-propanedione (PPD, Sigma Aldrich, St. Louis, MO, USA); double distilled water (Carl Roth, Karlsruhe, Germany); brain-heart-infusion-broth (Carl Roth GmbH + Co. KG, Germany) Nunc Thermanox coverslips, 13 mm diameter, were from Thermo-Fisher Scientific (Waltham, MA, USA). For bacterial adhesion testing, commercially purchased Enterococcus faecalis (ATCC 29212) (Leibniz Institute DSM, Braunschweig, Germany) was used.

### 2.2. Sample Coating

For the current study, a total of 24 commercially purchased sand-blasted-acid-etched (SLA) titanium samples (R:10 mm × 0.2 mm) of grade-4 (Ti-Pure-Plus^®^ BEGO Medical GmbH, Bremen, Germany) were used. The coating of 12 samples was conducted via photo-grafting of SBMA using soft UV light, as described in (Es-Souni et al, Materials & Design, 2019, 182, 108031). In brief: the sterile samples underwent a 5.5 min oxygen plasma treatment with subsequent TMSPMA vapor deposition (2 h at 70 °C) and curing (1 h 100 °C). Briefly, two solutions were prepared: An aqueous solution containing 28% (*w*/*w*) SBMA, and a 52 mM PPD initiator solution in 2-propanol. Following degassing of the monomer solution, the initiator was added in a molar ratio of 1 PPD:625 SBMA and covered with 0.35 mL of the SBMA/PPD-solution. After thoroughly cleaning in order to eliminate loosely bound species, the samples were stored dry at room temperature.

### 2.3. Examination of the Surface Morphology and Chemical Composition

Examination of the surface morphology and chemical composition prior and after pSBMA coating of samples were examined with the scanning electron microscope (SEM; Carl Zeiss AG, Dresden, Germany), equipped with an X-ray electron diffraction spectrometry (EDS; EDAX, Inc., Oxford, Great Britain) facility.

### 2.4. Water Contact Angle Measurement and Wettability

Water contact angle (WCA) measurements of the coated and as-received titanium species were performed using the sessile drop method (Data Physics Instruments GmbH, Berlin, Germany).

### 2.5. FTIR Analysis

Transmission spectra of coated and bare titanium samples were recorded using an attenuated Total Reflection—FTIR spectrometer (Spectrum, Perkin Elmer, Waltham, MA, USA) in the range of 4000–400 cm^−1^.

### 2.6. Bacterial Contamination of the Samples

**Figure 1:**
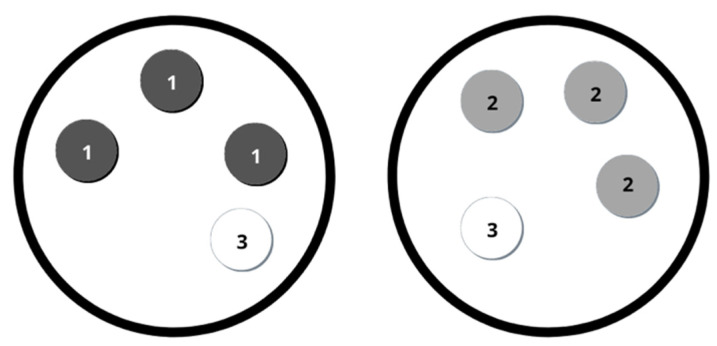
The study group consisted of three SPE coated titanium plates (1) and a single Thermanox™ plate. Three non-grafted titanium plates (2) and a single Thermanox™ plate (3) served as the control. Both dishes were incubated isochronously. All experiments have been performed in quadruplicate.

Bacterial colonization was comparatively assessed with coated and non-coated titanium samples (n: 12). Thermanox™ coverslips served as control (n: 8) (Figure 1). According to the experimental design, three coated samples and one Thermanox™ coverslip were placed in a single petri dish, whereas three uncoated Ti substrates and one Thermanox™ coverslip placed in a second dish served as the control. All samples were incubated with 10 mL brain-heart-infusion-broth and contaminated with 10 µL Enterococcus faecalis for 5 days at 37 °C in a humidified atmosphere of 95% air and 5% CO_2_. After two days, the medium was removed from the cell culture dishes and replaced with another 10 mL of BHI containing 10 µL E. faecalis. This process was repeated after another two days. On the fifth day, the samples were evaluated. Samples were washed gently with Dulbecco’s Phosphate buffered saline solution (PBS) to remove the loose bacteria prior to the assessment.

### 2.7. Fluorescence Microscopy (FM)

Antifouling properties of coated and bare titanium substrates as well as Thermanox coverslips were investigated using the Bac-Light, LIVE/DEAD™ Viability Kit staining (Thermo-Fisher Scientific, Waltham, MA, USA). The SYTO 9 green-fluorescent nucleic acid stain labels all bacteria in a population. The red-fluorescent nucleic acid stain propidium iodide penetrates only bacteria with damaged membranes and causes a color shift from green to orange/red, when both dyes are present. All specimens were placed in petri dishes, covered, and left incubated in the dark environment for 15 min at room temperature. Subsequently, the samples were washed with PBS, placed on glass slides, and examined using fluorescence microscopy (Axio Observer. Z1, and Axio Cam, MRc, Carl Zeiss, Jena, Germany) with image processing software (Axio Vision Rel. 4.8, Carl Zeiss Microscopy GmbH, Jena, Germany) to evaluate the bacterial colonization as well as viability of the biofilm. Each specimen was assessed in scores from 1 (no detectable bacteria) to 4 (measurable thick biofilm), as described by Acil et al. [18]. Classification of colonization was conducted in four degrees: (a) class 1: no microorganisms detectable; (b) class 2: sparse microorganisms; (c) class 3: many microorganisms and conglomerates; (d) class 4: dense microbial colonization. Fluorescence imaging analysis was conducted in quadruplicate.

### 2.8. Scanning Electron Microscopy

For the scanning electron microscope evaluation, coated and native titanium samples as well as Thermanox™ coverslips originating from five-day bacterial cultures were fixed in 3% glutaraldehyde solution for 2 h at 4 °C. Thereafter, the specimens were gently washed with PBS and placed on sample trays in a sample holder. The samples were dehydrated in an ascending ethanol series of 50–100% and wet with 1,1,1,3,3,3-hexamethyl disilazan, which replaced critical point drying. Thereafter, all specimens were sputtered with a 15 nm thick gold layer (Sputter-Coater SCD 500, BAL-TEC, Maidenhead, UK). SEM evaluation was performed with SEM XL 30CP (Philips Electron Optics GmbH, Kassel, Germany) at magnification of 500× to 5000×. For the analysis of the specimens, the same semi-quantitative scoring system was used as in the vital-fluorescence microscopy. The scale ranged from 1 (no detectable bacteria) to 4 (measurable thick biofilm). SEM analysis was conducted in quadruplicate.

### 2.9. Colony-Forming Units

The colony-forming unit (CFU) was used to estimate the number of viable bacteria. For the estimation of colony-forming units (CFU), BHI-agar plates were used. One coated Ti sample and one bare Ti substrate were placed in a 6-well plate with each containing 5 mL PBS. The 6-well plate was placed in an ultrasonic bath for 10 min in order to loosen the adherent bacteria. The bacterial suspension was transferred into a reaction vessel and diluted with NaCl in a ratio of 1:1000. The bacterial suspension was diluted in triplicate (1:100), whereas four dilutions were conducted on two BHI-agar plates (SPE-Ti and Ti) Both BHI-agar plates were incubated at 37 °C for 24 h. The CFU was quantified by using a germ counter. CFU experiments were conducted in quadruplicate.

### 2.10. Statistical Analysis

Descriptive statistical analysis was carried out using IBM, SPSS, Statistics version 24.0 for Windows (IBM GmbH, Ehningen, Germany). A paired t-test was conducted to evaluate the statistical differences between the two groups (pSBMA-Ti and Ti). The significance level was set to (*p* < 0.05). 

## 3. Results

### 3.1. Examination of the Surface Morphology and Chemical Composition

**Figure 2:**
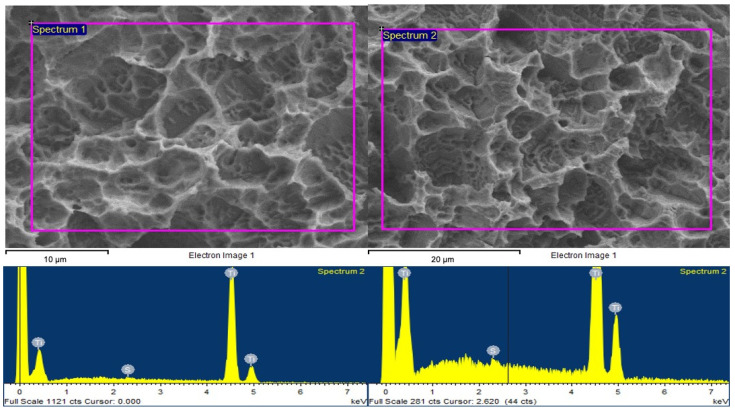
Visual data of spectrum analysis of Ti surfaces prior (**left**) and after (**right**) pSBMA grafting.

Table 1 showing the spectrum processing of Ti surfaces a. prior and b. after pSBMA grafting. Visual data of spectrum analysis of Ti surfaces prior and after pSBMA grafting were shown in Figure 2.

### 3.2. Water Contact Angle Measurements and Wettability

**Figure 3:**
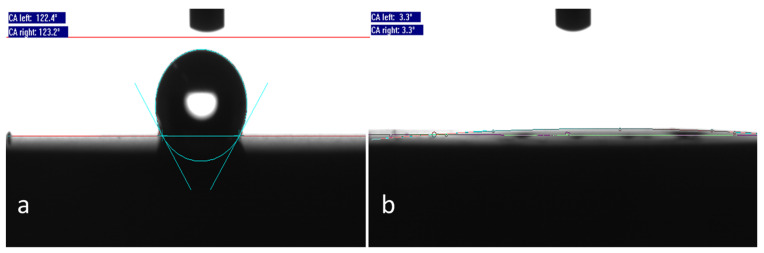
(**a**) Native, macro-roughened titanium surfaces exhibit a pronounced hydrophobic character (WCA = 123°), whereas (**b**) pSBMA coated ones are super-hydrophilic (WCA = 3°).

The wettability of coated and bare titanium surfaces is depicted in Figure 3. Native, macro-roughened titanium surfaces exhibit a pronounced hydrophobic character (WCA = 123°), whereas pSBMA coated ones are super-hydrophilic (WCA = 3°).

### 3.3. FTIR Analysis

**Figure 4:**
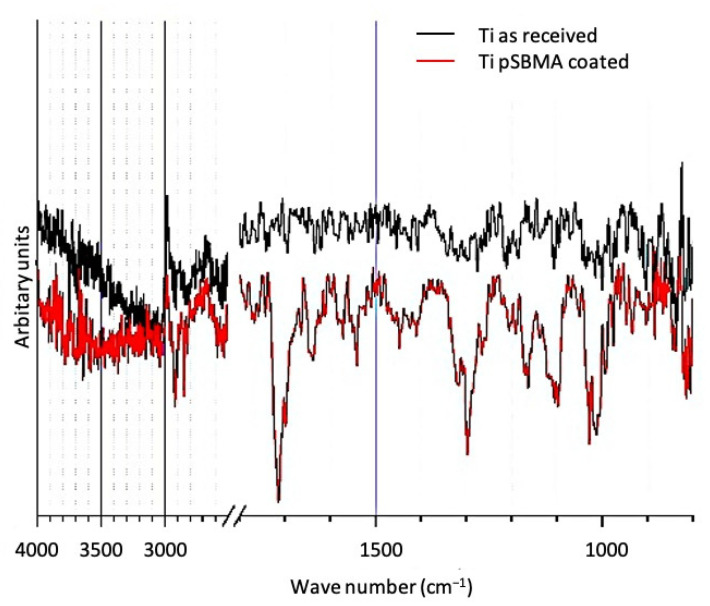
FTIR transmission spectra of a titanium surface in the as-received state and after pSBMA coating.

Figure 4 shows the FTIR transmission spectra of a pSBMA coated and a titanium sample in the as-received state. Compared to the featureless spectrum of the native surface, the pSBMA coated surface exhibit transmission bands that can be assigned to (1) C=O (1720 cm^−1^), S=O (1170 and 1090 cm^−1^), species characteristic to the pSBMA coating as well as (2) physisorbed water (3500 and 1640 cm^−1^).

### 3.4. Fluorescence Microscopy

**Figure 5:**
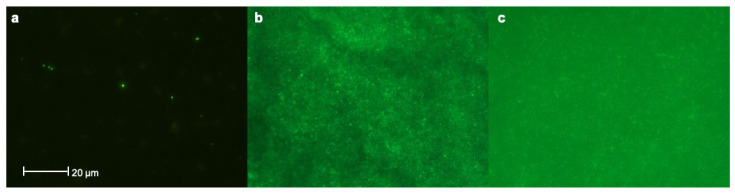
Assessment of bacterial colonization on samples via fluorescence imaging analysis revealed that; (**a**) Sparse microorganisms could be observed on pSBMA -Ti surfaces (class 2) (**b**) whereas Ti surfaces showed dense bacterial colonization (class 4) (**c**) Many microorganisms and conglomerates could be detected on Thermanox™ plates (class 3).

Assessment of bacterial colonization on samples via fluorescence imaging analysis revealed the presence of sparse microorganisms on pSBMA-Ti surfaces (class 2), whereas bare Ti surfaces showed dense bacterial colonization (class 4). Besides that, many microorganisms and conglomerates could be detected on Thermanox™ plates (class 3) (Figure 5).

### 3.5. Scanning Electron Microscopy

**Figure 6:**
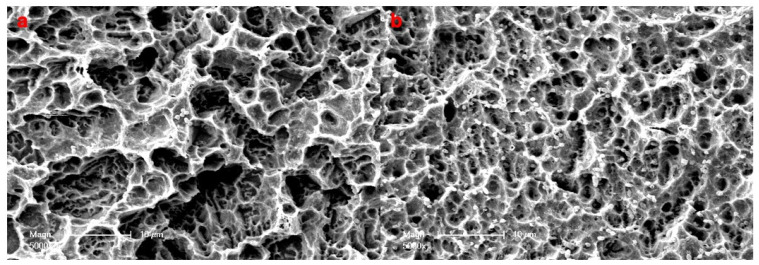
Semiquantitative assessment of bacterial colonization on samples via scanning electron microscopy (×5000) (**a**) Bacterial colonization on SPE-Ti surfaces revealed sparse microorganisms (class 2) (**b**) whereas Ti surfaces showed many microorganisms and conglomerates (class 3).

Semiquantitative assessment of bacterial colonization on samples via scanning electron microscopy at 5000 magnification showed sparse microorganisms on pSBMA—Ti surfaces (class 2) and many microorganisms and conglomerates on Ti surfaces (class 3) (Figure 6).

### 3.6. Colony Forming Units

**Figure 7:**
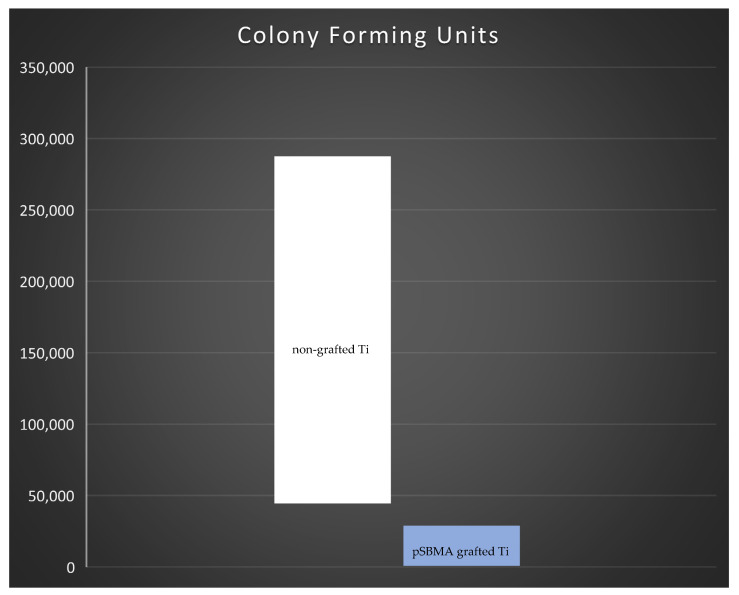
Statistical comparison of colony forming units between pSBMA grafted titanium (orange) and non-grafted titanium plates (blue) showed that the bacterial colonization non-grafted plates was higher compared to pSBMA grafted titanium surfaces. The difference was statistically significant (*p* = 0.021).

The statistical comparison of CFU between pSBMA grafted titanium and non-grafted titanium plates showed that the bacterial colonization on non-grafted plates was significantly higher compared to SPE grafted titanium surfaces (*p* = 0.021) (Figure 7).

## 4. Discussion

The use of implants has become a common treatment in several medical disciplines. Taking dentistry as an example, it appears that the increase in oral health awareness and improvement/ optimization of surgical procedures has led to a steadily increasing number of surgical interventions. Nonetheless microbial inflammation reactions may threaten treatment success. Modifying the surface morphology, wettability, and chemistry of an implant could represent promising solutions with respect to biofouling and related inflammatory reactions. The current study aimed to define a novel surface modification strategy to prevent bacterial adhesion—a crucial step for irreversible microbial attachment and further colonization—on titanium, the most widely used medical implant material. Among others, Enterococcus faecalis is known to participate in tooth root infections and to be able to remain in the surrounding bone after tooth extraction and colonize later implants [19,20]. Different strategies have been developed to encounter such biofouling, reaching from grafting leachable microbicides—such as antibiotics, antimicrobial peptides or ions—to creating so called “inert” coatings. The key prerequisite for biofilm formation is the step of adhesion and interaction of a species with the targeted surface. In this respect, it appears obvious that creating coatings with low interaction potential towards surrounding species might help to confer antifouling properties to implant surfaces. A promising approach is the use of biocompatible zwitterionic polymer coatings such as pSBMA [21,22,23]. It has been shown that in an aqueous environment containing solved ions, the zwitterionic nature of the coating is responsible for a hydration layer, where the structure of surrounding water molecules resembles that of bulk water. Such super-hydrophilic surfaces appear neutral, unfavorable to adsorption, and resist higher salt concentrations well [23].

In the present study, commercial sand-blasted-acid-etched titanium implant samples have been successfully coated with pSBMA—as shown with FTIR measurements—using a low-cost photo-polymerisation method. WCA measurements confirmed the super-hydrophilic nature of the coating. In vitro biofouling experiments in the presence of E. faecalis conducted in a qualitative manner (Fluorescence and SE microscopy) as well as in quantitative manner (CFU counts) revealed its antiadhesive nature. SEM and fluorescence microscopy showed both spares bacterial growth on the coated sample—probably due to coating failures such as scratches—compared to the dense bacterial film adhered on the bare titanium surface. Similar results were obtained for the CFU counts, where a statistically significant higher amount of CFU was noted on titanium in the as-received state.

Taken together, the present work is consistent with other studies already published in the field of antiadhesive zwitterionic coatings and offer a versatile and low-cost coating method.

It is very well known that, not only surface roughness but also surface wettability may influence bacterial adhesion to the implant surface [24]. The results of the current study revealed that, despite their higher roughness characteristics compared to non-grafted titanium surfaces, pSBMA coated titanium surfaces represented a significantly superior hydrophilicity which has determined the superior antifouling properties of SPE grafted titanium plates. The contact angle on the pSBMA grafted titanium surfaces could have resulted in super-hydrophilic properties as described by Wenzel [25]. Additionally, the superhydrophilicity of pSBMA grafted titanium surfaces could also be attributed to the interaction of adjacent polymer chains [26,27]. High packing density of pSBMA via photopolymerization processing described herein could have contributed to forming a uniform polymer coating and consequently helped achieve better hydrophilicity as previously suggested in the literature [28].

The results of the current experimental study showed that pSBMA coating of titanium surfaces could be a promising strategy in preventing bacterial adhesion. From a clinical point of view, infections around dental implants pose a great challenge for dental professionals, thus plaque accumulation and concomitant bacterial adhesion could lead to infections and implant loss. The surface modification technique described herein might offer a novel solution in preventing peri-implantary infections in the future. However, it should be kept in mind that improving the anti-adhesive properties of titanium surfaces by using polymers could also prevent the interaction of the implant surface both with bacterial cells and native cells at the implant recipient site. Preventing the attachment of proteins belonging to the physiological bone regeneration and/or osseointegration process could result in inadequate or insufficient integration of the implant [29]. In order to overcome this negative effect, promotion of osteo-induction via tethering of biologically active proteins such as bone morphogenetic proteins (BMP-2 and BMP-7) on the implant surface has been also reported [29,30]. Therefore, the osteoblast proliferation and/or osseointegration process on SPE grafted titanium surfaces warrants further research.

Similarly, the stability of the grafted polymers on the implant surface in situ remains of concern. An insufficient bonding strength between the coating and the implant surface could lead to degradation or separation of the coating, which might result in a more favorable environment for plaque accumulation or bacterial adhesion.

## 5. Conclusions

pSBMA coatings via photopolymerization of titanium surfaces seems to be a promising antiadhesion strategy, which should bring substantial benefits once certain aspects such as biodegradation and osseointegration were addressed. Additionally, commercial SAL-titanium substrates may be coated with the super-hydrophilic coating, appearing resistant to physiological salt concentrations and most importantly lowering E. faecalis colonization significantly, compared to titanium substrates in the as-received state. It appears very likely that pSBMA coatings may also prevent the adhesion of other germs.

## Figures and Tables

**Table 1:** Spectrum processing of Ti surfaces prior and after pSBMA grafting.

Element	Weight (%)	Atomic (%)
SK	0.25	0.37
TiK	99.75	99.63
Totals	100.00	

Spectrum processing: No peaks omitted. Processing option: All elements analyzed (Normalised). Number of iterations = 1. Standard: S—FeS2—1-Jun-1999 12:00 a.m. Ti—Ti—1-Jun-1999 12:00 a.m.

## Data Availability

Data supporting results could be found at the research laboratories of Christian-Albrecht University, Department of Oral and Maxillofacial Surgery.
